# Prevalence and patterns of combat sport related maxillofacial injuries

**DOI:** 10.4103/0974-2700.70744

**Published:** 2010

**Authors:** Gholamreza Shirani, Mohammad Hosein Kalantar Motamedi, Alireza Ashuri, Pooyan Sadr Eshkevari

**Affiliations:** Department of OMFS, School of Dentistry, Tehran University of Medical Sciences, Tehran, Iran; 1 Department of Oral and Maxillofacial Surgery, Trauma Research Center, Baqiyatallah University of Medical Sciences; and Azad University of Medical Sciences, Tehran; 2 Private Practice Dentistry, Tehran

**Keywords:** Maxillofacial trauma, combat sports, injury

## Abstract

**Aim:**

This study was designed to assess the prevalence, distribution, and patterns of injury among athletes engaged in combat sports and compare the prevalence, pattern, and types of oral and maxillofacial trauma in these athletes.

**Materials and Methods:**

A total of 120 male athletes engaged in four combat sports (boxing, taekwondo, kickboxing, and Muay Thai) who had sustained bodily trauma were studied; 95 subjects with at least one traumatic injury to the face requiring treatment were referred to us by the physician team. The type of injury (facial laceration, facial fractures, jaw dislocation, etc.), site of facial injury (jaw, nose, malar bone, teeth, etc.), dental injuries (tooth fracture, displacement, luxation, and avulsion), causative sport (boxing, taekwondo, kickboxing, and Muay Thai) as well as demographic data were recorded. Injuries were examined clinically and radiographically, and treated accordingly by a specialist. Treatment data and demographics were recorded for each subject. Recorded data were assessed, and χ^2^, ANOVA, and Kruskal–Wallis tests were used to statistically analyze and compare the data.

**Results:**

Of 120 subjects, 95 male subjects (79.2%), aged 18–25 years (avg. 20 years), had at least one traumatic injury to the face requiring medical treatment. These injuries included facial laceration, bone fractures (nose, mandible, and zygoma), dental injuries (displacement, luxation, fracture, and avulsion), and mandibular dislocation which were recorded in 83 (69.2%), 55 (45.1%), 53 (44.2%), and 8 (6.7%) cases respectively. Statistically significant differences were encountered among various injuries and the sports; kickboxing caused the most maxillofacial injuries and was identified as more injurious. Tooth fractures (59.7%) were the most common dental injuries, and the nose (84.7%) was the most frequently fractured facial bone. Lacerations were more common in Thai-boxers (93.3%). Injuries were significantly greater in professional rather than amateur athletes.

**Conclusion:**

In this study, prevalence of facial injuries from combat sports professionals was significantly high (roughly 80%), especially in kickboxing (in part due to use of less protective gear). Because the nose and teeth sustained the most injuries, they require more attention with regard to prevention. Kickboxing was the most injurious of these combat sports and caused the most significant number of maxillofacial trauma. More safety apparel and protective guards seem warranted in athletes of combat sports if facial injury is to be prevented.

## INTRODUCTION

Facial trauma is a part of combat sports and martial arts. Oral and maxillofacial injuries are thus common in athletes engaged in these sports. Trauma from sports is increasing due to the increasing trend among people toward exercise and physical fitness.[1–6] Varying trauma patterns result from different sports and have been reported in the literature; soccer, biking, and rugby are stated to be the most traumatic.[3,6–11] In view of the growing trend toward violence and consequent increase in altercations, there is a growing trend toward taking-up combat sports, especially in the younger age groups. Although injuries in the maxillofacial area from combat sports are less than soccer and rugby, the aggressive offensive/defensive nature of these sports, which necessitates striking and blocking various parts of the body and the face with techniques executed at full force and a minimal amount of protective gear, there is a high risk of sustaining dangerous injuries to the face.[2,4,12–14] There have been various studies on the different types of trauma to the craniofacial region throughout the literature. However, very few have addressed the prevalence, distribution, and pattern of trauma to the maxillofacial region from combat sports. Comparison of data from different combat sports and different societies can vary, assessment of which is the first step toward prevention. This was done to see which sport was more injurious and to assess the pattern of injury.

## MATERIALS AND METHODS

During the study period (2005–2009), 120 male athletes, who had sustained trauma from four various combat sports (boxing, taekwondo, kickboxing, and Muay Thai), were examined by the physician team. Those who had sustained maxillofacial trauma were referred to one maxillofacial surgeon for assessment of maxillofacial injuries; 95 subjects who had at least one traumatic injury to the face requiring medical treatment were included in this study. The remaining 25 had body injuries only and were thus excluded. These were unintentional injuries during training and competition. The maxillofacial injury was examined both clinically and radiographically (panoramic and posterior anterior views for the mandible, water’s view, and submentovertex for the zygoma, lateral views for nasal fractures). CT scan was done when indicated (i.e., zygomatic fractures) and treated accordingly (repair of lacerations, hospital admission, and open or closed reduction of jaw or nasal fractures). The data relative to the injury type and the sport as well as demographic data were documented in the hospital records of the patients and remained confidential. The data collected (by the maxillofacial surgeon) from the patient files included the type of injury (facial laceration, facial fractures, jaw dislocation, etc.), site of facial injury (jaw, nose, malar bone, teeth, etc.), dental injury (tooth fracture, mobility, luxation, and avulsion), causative sport (boxing, taekwondo, kickboxing, and Muay Thai), and demographic data.

Data analysis was done using the χ^2^, ANOVA, and Kruskal–Wallis tests to statistically analyze the obtained data. Use of data from patient files and treatment data without disclosure of names or patient identification and with the prior written consent from the patients was granted approval by our ethics committee.

## RESULTS

A total of 120 male athletes had sustained body injuries from four combat sports (boxing, taekwondo, kickboxing, and Muay Thai).

### Pattern

Of 120 subjects, 95 (79.2%), aged 18–25 (avg. 20 years), had at one or more facial injuries; 25 (20.8%) had body injuries only and they were excluded. Facial lacerations, bone fractures, dental injuries, and mandibular dislocation were recorded in 83 (69.2%), 55 (45.1%), 53 (44.2%), and 8 (6.7%) cases, respectively. Statistically significant differences were encountered among various injuries sustained and the sport kickboxing caused the most maxillofacial injuries and was more traumatic.

Facial lacerations requiring sutures were seen in 83 athletes (69.2%); the majority was Thai boxers (93.3%). Facial lacerations were treated and closed in layers with resorbable sutures beneath the skin and fine nylon sutures subcutaneously.

Eight subjects (6.7%) sustained mandibular dislocations (three Thai boxers, three taekwondo athletes, and two kickboxers) which were reduced manually on site by the team doctor.

**Figure 1:**
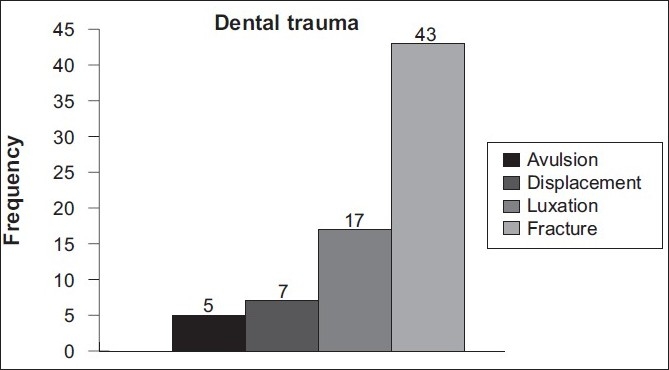
Type of dental injury in athletes of 4 combat sports

Forty-three (59.7%) patients had some form of dental trauma (fracture, avulsion, displacement, or luxation). Tooth luxation following direct trauma was seen in 17 (23.6%), whereas tooth displacement and avulsion were seen in 7 (9.7%) and 5 (7%) athletes, respectively [Figure 1]. All dental traumas involved the anterior teeth.

**Figure 2:**
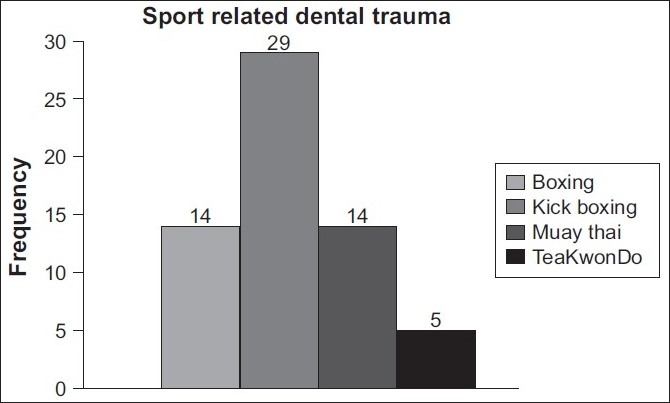
Frequency of dental trauma in athletes of 4 combat sports

Of the 53 athletes (44.2%) who had sustained dental trauma, kickboxers comprised the majority [Figure 2]. Dental trauma in kickboxers was (*n* = 20) 66.7%, Thai boxers (*n* = 14) 46.7%, boxers (*n* = 14) 46.7%, and Taekwondo athletes (*n* = 5) 16.7%.

Maxillofacial fractures from kickboxing, Muay Thai, boxing, and taekwondo resulted in 29, 21, 18, and 5 injuries, respectively. Fractures were seen in 55 (45.1%) athletes. Most (73.3%) of kickboxers had facial fractures (*n* = 22) followed by Thai boxers (*n* = 15, 50%), boxers (*n* = 14, 46.7%), and taekwondo athletes (*n* = 4, 13.3%). These values were statistically significant (*P* < 0.05).

**Figure 3:**
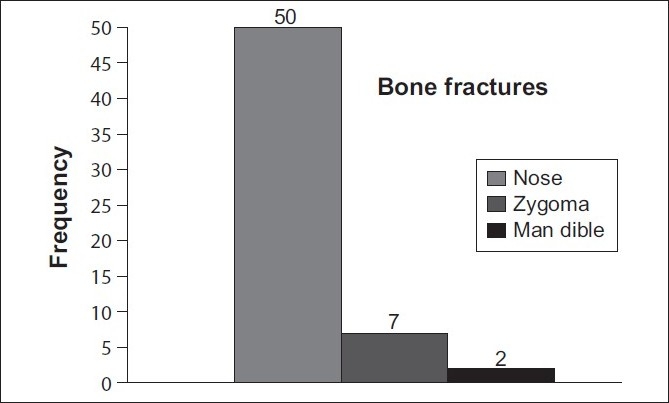
Frequency of maxillofacial fractures in athletes of 4 combat sports

Nasal, zygomatic, and mandibular bones comprised the most frequently fractured facial bones accounting for 84.7% (50), 12% (7), and 3.3% (2) of the cases, respectively [Figure 3]. Maxillofacial injuries were more common among professionals compared to amateurs (86% and 42.1%, respectively). The difference was statistically significant (*P* = 0.59).

## DISCUSSION

A review of the literature on the various types of sport-related trauma revealed that craniomaxillofacial injuries are of the most common.[3,4,8,9,14] Despite the high incidence however, craniomaxillofacial trauma from martial arts has received less attention than would be expected. This could be due to the importance of other organ injuries, which may impair more important functions of an athlete requiring more attention.[8] With regard to facial injuries, the most frequently reported maxillofacial bones fractured involve the nasal, zygomatic, and mandibular bone from contact sports, such as soccer and rugby, as a consequence of an impact from another player.[1,6,11] The prevalence of facial injuries is high in combat sports and the martial arts as well.[2,4,9]

In the prevalence of injuries, facial laceration was found to be the most common injury in our study (*n* = 83, 69.2%). This was similar to several other studies.[2,4,9] In our subjects, facial bone fractures (*n* = 55, 45.1%), dental trauma (*n* = 53, 44.2%), and dislocation of the mandible (*n* = 8, 6.7%) were prominent findings. Kickboxing was found to be the most significant cause of facial and jaw fracture injuries. The majority of the subjects, 22 (73.3%), suffering maxillofacial fractures were kickboxers. Tooth fractures (59.7%) were the most common type of dental injury, and the nasal bone (84.7%) was the most commonly fractured bone obviously, because it is not protected by face guards. Nasal fractures accounted for 50 (84.7%) of fractures. The majority of sports-related craniomaxillofacial injuries, however, are fortunately of a minor nature causing lacerations followed by dentoalveolar fractures and minor facial bone fractures.[3,4,8,9,14] Facial lacerations requiring sutures were seen in 83 athletes (69.2%); a significant number of which were Thai boxers (93.3%). This may be due to the fact that this sport targets the face with less protective gear. However, kickboxing was found to be more injurious than the other combat sports with regard to facial and dental fractures because more force is applied by kicks to the jaws rather than punches. Four types of dental injuries (tooth fracture, displacement, luxation, and avulsion) were focused prominently in our study. Tooth fracture was the most common type of dental injury observed.

Dental trauma from blows to the jaws may result in tooth fractures (and pulpal exposure and pain requiring endodontics) or tooth avulsion; both are considered dental emergencies. An avulsed tooth must be immediately placed back into the socket if it is to take; if this is not performed within 30 min it will develop root resorption and exfoliate within several years. Jaw fractures such as condyle fractures also need immediate attention; trauma to the mandibular condyle may lead to mandible undergrowth, overgrowth, or ankylosis years later not to mention mandibular asymmetry.

The importance of this study lies in the fact that it is the first of its kind in Iran. Thus, it provides relevant data, which can be used for further research and comparisons subsequently. It also points out and documents the dangers involved in taking-up such sports and stresses the need for safer rules and regulation, and also for better protective gear. These issues may be reflected to influence the Chairman of the Olympic Committee or the Martial Arts Federation.

Limitations of the study include lack of assessment of more parameters and lack of follow-up of many of the patients for assessment of treatment or to assess late complications. Athletes sustaining trauma are known to continue the competition bout, despite injury and thus worsening it. Not referring for diagnosis or treatment may pose flaws in data collection; many aspects were not assessed. Additionally, our study scope was limited and thus, does not address post-treatment complications, disabilities, loss of athletic performance, decreased facial esthetics, problems with occlusion, loss of jaw function, etc. Moreover, the impact of these injuries have both physical and psychological effects on the performance of the athletes, their career, and quality of life.

## CONCLUSION

Prevalence of facial injuries from trainees in combat sports was high (roughly 80%) in this study. Injuries were significantly higher in professional athletes (in part due to use of less protective gear). The nose and teeth sustained the most injuries and thus, require more attention with regard to prevention. Kickboxing was the most injurious combat sport and caused the most significant number of maxillofacial injuries. More safety apparel and protective guards seem warranted in athletes of these combat sports if facial injury is to be prevented.
